# In Vivo Glycemic Response of Fruit-Based Mango (*Mangifera indica*) and Pineapple (*Ananas comosus*) Bars in In Vitro and In Silico Enzyme Inhibitory Effects Studies

**DOI:** 10.3390/foods13142258

**Published:** 2024-07-17

**Authors:** Yolanda E. Pérez-Beltrán, Abraham Wall-Medrano, Monserrat A. Valencia Estrada, Jorge A. Sánchez-Burgos, Francisco Javier Blancas-Benítez, Juscelino Tovar, Sonia G. Sáyago-Ayerdi

**Affiliations:** 1 Laboratorio Integral de Investigación en Alimentos, Tecnológico Nacional de México/Instituto Tecnológico de Tepic, Nayarit 63175, Mexico; yoelperezbe@ittepic.edu.mx (Y.E.P.-B.); moaivalenciaes@ittepic.edu.mx (M.A.V.E.); jsanchezb@ittepic.edu.mx (J.A.S.-B.); fblancas@ittepic.edu.mx (F.J.B.-B.); 2 Instituto de Ciencias Biomédicas, Universidad Autónoma de Ciudad Juárez, Chihuahua 32310, Mexico; awall@uacj.mx; 3 Division of Food and Pharma, Lund University, 22100 Lund, Sweden; juscelino.tovar@food.lth.se

**Keywords:** mango, pineapple, healthy snacks, bioactive compounds, glycemic response, enzyme inhibition, molecular coupling

## Abstract

The habitual consumption of snacks has the potential to enrich or harm the diet. They can contribute to excessive caloric intake and hyperglycemia. Thus, there is an increasing interest in snacks with health-promoting properties. This study aimed to demonstrate the beneficial effect of two fruit-based bars on glucose levels through in vitro, in vivo, and in silico assays. Mango (*Mangifera indica* L.) and pineapple (*Ananas comosus* L.) bars (MB and PB) were prepared, and chemical composition, postprandial glycemic response, glycemic index (GI), and glycemic load (GL) were evaluated. The inhibitory effect of fruit bar extracts on α-amylase and α-glucosidase activity and their respective molecular docking was assessed. MB and PB showed the lowest postprandial glycemic response vs. the control bar (*p* < 0.005), a lower GI (CB: 64.20, PB: 53.20, MB: 40.40), and a GL of 10.9 (CB), 7.9 (PB), and 6.1 (MB), (*p* < 0.05). MB and PB showed the highest inhibition % of α-amylase (61.44 and 59.37%, respectively) and α-glucosidase (64.97 and 64.57%). Naringenin (−1692.5985 and −2757.674 kcal/mol) and ferulic acid (−1692.8904 and −2760.3513 kcal/mol) exhibited more favorable interaction energies against α-amylase and α-glucosidase activity. The presence of polyphenols from the fruit influenced enzymatic inhibition. Likewise, the dietary fiber in the bars evaluated allowed us to observe a positive effect that favors glycemic control, making them a healthy alternative for snacking.

## 1. Introduction

Current trends in food consumption are characterized by the ingestion of snacks, which play an essential role in providing energy sustenance between main meals [1]. The European continent and Australia stand out for their high snack consumption. Likewise, countries like the United States, followed by Mexico and China, have increased the consumption of snacks rich in sugar and saturated fats [2,3].

In this sense, when consumers consciously choose snacks, they can enrich their diet by supplying essential nutrients and bioactive compounds beyond calories. However, if consumers choose incorrectly, especially ultra-processed snacks with high saturated or *trans*-fat content, artificial additives, sugar, and therefore a high glycemic index, it can contribute to the development of several metabolic diseases such as overweight, obesity, dyslipidemia, hyperglycemia, and insulin resistance [4,5].

On this basis, it is highlighted that Type 2 Diabetes Mellitus (T2DM) has steadily increased in terms of prevalence, incidence, disability-adjusted life-years, and mortality [6]. Fortunately, the rising public awareness of this disease has promoted people’s adherence to primary (e.g., healthy eating practices) and secondary prevention (e.g., self-care disease management). Programs and a well-established food innovation pipeline aim at preventing and/or controlling postprandial hyperglycemia [7]. In this sense, monitoring some indices, such as glycemic load (GL), glycemic index (GI), and glycemic response (GR), that are used to rank carbohydrates in foods can assess their impact on postprandial glucose [8].

Currently, the global diabetic food market is valued at USD 7.4 billion, and it will reach USD 10.4 billion by 2028 at a compound annual growth rate of 4.2% [9]. Most in-market diabetic foods are low-calorie/low-sugar goods aimed to control hyperglycemia, and food technologists have focused on developing ready-to-eat baked and confectionery goods [10,11]. Food choices labeled as “smart-carbs” or “smart GI (glycemic index)” are gaining similar recognition to “high fiber” or “whole grain” labeled foods as healthy nutrition options to avoid T2DM and its premorbid condition (pre-diabetes). Novel foods prepared with resistant starches, inhibitors of polysaccharide-hydrolases, and mono/di-saccharide transporters are emerging within this market [12]; the challenge for developing this type of product lies in identifying food components capable of modifying the enteral/systemic metabolism of carbohydrates to support health claims and product differentiation in this exceptionally competitive market segment. 

In this way, certain tropical fruits, such as mango (*Mangifera indica* L.) and pineapple (*Ananas comosus* L.), have a unique phytochemical profile with anti-diabetic potential [13]. They contain many highly bioaccessible and fermentable phenolic compounds and antioxidant dietary fiber. Phenolic compounds have received particular interest due to their biological activity; it has been reported that this type of compound plays a vital role in modulating the activity of digestive enzymes such as α-glucosidase and α-amylase, which are responsible for the hydrolysis of carbohydrates [14,15]. In preceding reports, the main bioaccessible phenolic compounds identified in mango bars were gallic acid, caffeic acid, mangiferin, or flavonoids such as kaempferol, and quercetin, and in pineapple bars they included vanillic, syringic, chlorogenic, caffeic, and ferulic acids, flavonoids such as quercetin and kaempferol, among others [16,17]. 

However, one of the most significant gaps in the field is the need for studies on the post-intake behavior and impact of snacks since most studies only deal with final product characterization. Considering this context, the relevance of the present study is highlighted, as it contemplates a more extensive evaluation of food products with a potential effect on health with in vitro, in silico, and in vivo techniques.

This research aims to demonstrate the in vivo glycemic response to the consumption of fruit-based mango (*Mangifera indica* L.) and pineapple (*Ananas comosus* L.) bars and the in vitro and in silico enzyme inhibitory effect.

## 2. Materials and Methods

### 2.1. Preparation of Fruit Bars

Mango “Ataulfo” and pineapple “Esmeralda” fruits were purchased in a local market (Nayarabastos, Tepic, Nayarit, Mexico). The fruits were later transported, disinfected, and treated to obtain fruit bars (simple molding without additives), according to Hernández-Maldonado et al. [17] at the Technological Institute of Tepic. As a control bar, a known commercial fruit bar with mango declared the ingredients used (58 g portion: 180 kcal, 7 g total fats, 29 g carbohydrates, 3 g dietary fiber, 12 g sugar, and 2 g protein). It is important to mention there are no similar snack bars with only fruit in the market (in Mexico). Some bars were separated for glycemic index (GI) evaluation. The remaining bars were lyophilized for 24 h (FreeZone 6, Labconco, Kansas City, MO, USA), ground (NutriBullet, NBR-0804B, Los Angeles, CA, USA), sieved (5 mm), and stored at −20 °C until further analysis.

### 2.2. Chemical Composition of Mango “Ataulfo” and Pineapple “Esmeralda” Bar 

Fruit bar samples (MB and PB) were subjected to proximate analysis using the methods of AOAC [18], moisture (Method 925.10), crude fat (Method 920.39), protein (Method 920.87), and ash (Method 923.03). The phenol-sulfuric method evaluated total carbohydrates [19]. Dietary fiber (DF) was analyzed by the AOAC’s [18] enzymatic–gravimetric method (Method 991.42) modified by Mañas and SauraCalixto [20]. For the determination of total soluble polyphenols (TSPs), an aqueous-organic extraction was carried out according to the methodology proposed by Pérez-Jiménez, Arranz, and Saura Calixto [21]. The Montreau [22] method determined TSP in extracts with modifications. The results were expressed in gallic acid equivalents (GAE; mg/g dry mass) using a standard curve of gallic acid (y = 6.7937x − 0.0413, R^2^ = 0.9874).

### 2.3. Glycemic Response In Vivo Study

The experimental strategy used in this study followed the methodology proposed by Bellmann et al. [23] to evaluate the human glycemic response. 

Thirteen young participants (20–30 years old) were enrolled in a randomized, controlled, double-blind crossover trial. The in vivo study was conducted in the Clinical Laboratory of the Department of Health Sciences of the University of Ciudad Juarez. Specialized personnel from the clinic collected the data from participants and samples. The inclusion criteria were stable body weight in the last month (21 < Body Mass Index < 28.9); exclusion criteria were pregnancy, fasting glycemia ≥ 100 mg/dL (5.6 mM/L), the presence of chronic and metabolic diseases, use of over-the-counter (OTC) drugs, and the ingestion of nutritional supplements affecting glycemic response at least six months before participation. The experimental protocol was approved by the Autonomous University of Ciudad Juarez (UACJ) ethics board (CIEB-2019-1-075) and was conducted according to the Helsinki Declaration. Informed consent was obtained from all participants before enrollment (Figure S1). 

Before intervention sessions, participants were instructed to avoid strenuous physical activity for 72 h before any test day and to fast at least 12 h before arrival at the laboratory. Each participant attended four sessions (the first was informative, and the second to fourth were experimental sessions), spaced by 2 days (washout period), and underwent a 24 h food recall to evaluate any potential bias in glycemic response unrelated to the intervention.

In experimental sessions, trained nurses took a finger-prick capillary blood sample from participants who had fasted for 8 h. The subjects randomly received a portion of any of the tested fruit bars [control (C), mango (MB), and pineapple (PB) bars] corresponding to 50 g of available carbohydrates [total carbohydrates (by difference)—dietary fiber] [24] and instructed to consume it within 10 min under the observation of a qualified staff member. In every food challenge, the glycemic response at 0 (fasting), 15, 30, 45, 60, 90, and 120 min was tracked using a blood glucose measuring device (ReliOn^®^ PRIME blood glucose monitoring system, Wal-Mart Stores, Inc, Bentonville, AR, USA). The randomization schedule (permuted blocks by enrollment date/time) was supervised only by a team member and blinded to other team members and participants (double-blinded). During the test period, participants remained seated. 

### 2.4. Glycemic Index (GI) and Glycemic Load (GL)

The 120 min (2 h) recorded glycemic values for each tested sample (C, MB, PB) were used to calculate the corresponding area under the curve (AUC) using the trapezoid method [25,26], and any area falling beneath the initial fasting glucose concentration was not included in the calculation [24]. The AUC of a drink (500 mL) containing 50 g of anhydrous glucose was used as the reference food for GI calculations [27]. GI and glycemic load (GL) values were further calculated [10,24].

### 2.5. Enzyme Inhibition 

α–amylase activity: The inhibitory potential of ethanolic extracts of mango (MB) and pineapple (PB) bars for digestive enzymes was evaluated following the methodology of Granfeldt, Björck, Drews, and Tovar [28], with some modifications. Acarbose and gallic acid were also used as a standard to evaluate the inhibition percentage. Briefly, 4% starch was suspended and gelatinized in 5 mL of distilled water, 2.5 U/µL α-amylase enzyme (Sigma, A3176 Co., St. Louis, MI, USA) was suspended in 0.02 M sodium phosphate buffer at pH 6.9, and 100 µL of the sample was added. The mixture reaction was carried out in a shaking bath at 37 °C for 60 min, after which 4 mL of 0.1 M NaOH was added to stop the reaction. The current mixture went through a centrifugation process at 3000 rpm for 5 min. The supernatant was collected in 10 mL with a buffer. The volumetric solution was taken to determine the glucose content using the GOD-POD; it was incubated at 37 °C for 15 min. The sample was read in a microplate reader (Biotek^®^, Synergy HT, Winooski, VT, USA) at 505 nm. 

The enzyme activity was determined with Equation (1):

(1) $$I\%=\frac{\left(Abs\;blank-Abs\;sample\right)}{Abs\;blank}*100$$

α-glucosidase activity: The inhibition of α-glucosidase in MB and PB was evaluated using an ethanolic extract following the methodology described by Nair, Kavrekar, and Mishra [29], to which 25 μL of sample and 100 μL of glucosidase solution (0.19 U/μL) (pH 6.9), (Sigma, G5003 Co., St. Louis, MI, USA) were added and pre-incubated at 37° C for 10 min. Then, 25 µL of a solution of *p*-nitrophenyl-α-D-glucopyranoside (13, 23, and 33 mM) in 0.1 M phosphate buffer (pH 6.9) was added to each well. The reaction mixtures were incubated at 37 °C for 60 min. The absorbance was read at 410 nm and compared to a control with 25 μL buffer instead of the extract using the Synergy HT microplate reader (Biotek^®^, Synergy HT, Winooski, VT, USA). The enzymatic activity was calculated using Equation (1).

### 2.6. In Silico Assay for α-Amylase and α-Glucosidase

The ligand (phenolic compound) was obtained from the PubChem chemical molecule database (https://pubchem.ncbi.nlm.nih.gov/ accessed on 20 March 2022), and the molecular coupling test was carried out between the following phenolic compounds: ferulic acid (CID 445858), butein (CID 5281222), catechin (CID 9064), naringenin (CID 932), quercetin (CID 5280343) and kaempferol (CID 5280863). The 3D structure of α-amylase and α-glucosidase (targets) in PDB format were obtained from the Protein Data Bank protein database (https://www.rcsb.org/ accessed on 20 March 2022). According to the Universal Protein Resource (UniProt), the enzymatic identification for α-amylase α-glucosidase is 1VAH and 3WY1, respectively. The energy minimization of ligands and enzymes was carried out with the principle of molecular mechanics using the Hartree-Fock algorithm and protocols in Discovery studio visualizer (Ver. 21.1.0.0) [30] and UCSF Chimera (Ver. 1.14) [31]. Once the calculation was finished, the final structure was saved in mol2 format for later manipulation. Finally, after obtaining the target proteins and ligands, the Swiss Bioinformatics Institute (SIB) website service performed and optimized the coupling procedure [30,32]. The results of the molecular coupling were visualized in USCF Chimera and evaluated using the FullFitness parameter (FF, spontaneity of the formation of the enzyme-ligand complex), calculated as the average of 30% of the “*n*” most favorable energies of a cluster and used to reduce the risk of few complexes that penalizes an entire cluster. Subsequently, the cluster with the most favorable FF was used to determine the residues generating the complex. 

### 2.7. Statistical Analysis

The GI and GL data obtained were analyzed using the Kruskal–Wallis test, with a significance value of *p* < 0.05. All results are expressed as the mean ± standard deviation. Analysis of variance (ANOVA) was used. To evaluate the significant differences between the treatments, a Fisher LSD test was used, with a confidence level of 95%, using the STATISTICA version 12 program.

## 3. Results

### 3.1. Chemical Composition of Mango and Pineapple Bars 

**Figure 1:**
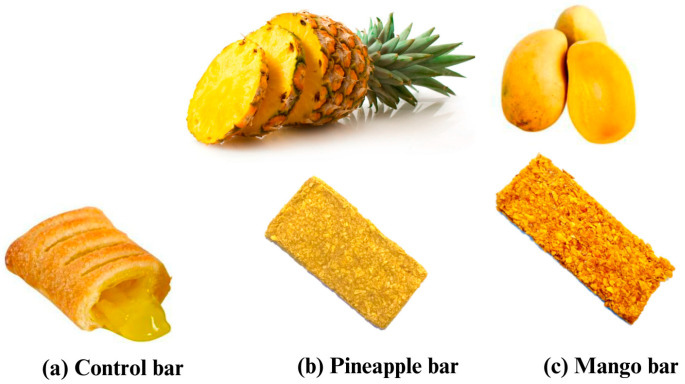
Graphic representation of the bars used in this study.

Table 1 shows the results obtained from the nutritional composition of mango (MB), pineapple (PB), and control bars (CB) for a portion size of 30 g, and in Figure 1, the appearance of the bars used is represented. In determining moisture, values with significant differences were obtained, with MB being the one that presented the lowest moisture percentage (10.23 ± 0.03%), with values ranging from 10 to 13%. In this case, values of 10% or less are associated with low water activity, indicating greater product stability. In the evaluation of ashes, the results showed significant differences between the three samples, with CB being the one that represents the lowest content in ashes (1.24%). Significant differences between all the samples for proteins were observed: PB (2.73 ± 0.04%) showed the highest protein content, followed by MB (2.41 ± 0.06%), and finally, CB (1.63 ± 0.04%). Regarding lipids, the CB showed the highest content (4.64 ± 0.04 %), which was attributed to incorporating vegetable oil, margarine, and vegetable fat declared in ingredients, unlike MB and PB, where only dried fruit was used. For carbohydrates (CHO’s g/30 g DW) content, no significant differences were found between MB and PB (15.06 ± 0.2 g/30 g DW and 14.88 ± 0.2 g/30 g DW), but CB showed the highest content (17.01 ± 0.1 g/30 g DW). In the case of the total dietary fiber (TDF g/30 gDW), significant differences were found between the three samples, highlighting a higher TDF content in fruit bars (MB: 9.55 ± 0.2 g. and PB: 7.37 ± 0.3/30 g) compared to CB with only 1.96 ± 0.1 g in the portion size. 

Finally, for total soluble polyphenols (TSP), significant differences were found between the three samples; in this case, MB showed the highest content (46.47 ± 0.1 mg GAE/g), followed by PB (14.90 ± 0.0 mg GAE/g), and CB presented the lowest content (5.01 ± 0.2 mg GAE/g). The presence of DF and polyphenols in the samples improves the functional properties of the bars.

### 3.2. Glycemic Response In Vivo Assays: Glycemic Index (GI) and Load (GL)

Thirteen (*n* = 13) individuals were enrolled to participate in this trial; 23% (*n* = 3) were men, and 77% were women (*n* = 10). The average age of the participants was 22.3 ± 5 years old, and the mean Body Mass Index (BMI) was 24.78 ± 2.8 kg/m^2^.

**Figure 2:**
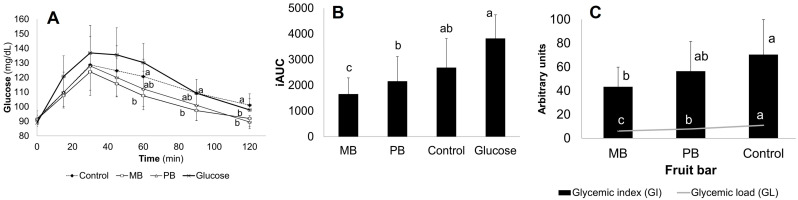
Postprandial glycemic response. (**A**) Glucose levels after ingesting fruit-based bars, control bar, and glucose solution. (**B**) Area under the curve of tested samples. (**C**) Glycemic index and glycemic load of mango, pineapple, and control bar. Lowercase letters (a–c) indicate significant differences (*p* < 0.05).

Figure 2A shows the postprandial glycemic response, which indicates the glucose levels in the bloodstream after ingesting CB, MB, PB, and glucose during the 120 min evaluation. It was observed that the time-trend differences in glycemic curves between assayed samples occurred between 60 and 120 min. On the other hand, the iAUC for MB and PB, to a lesser extent, was significantly lower than that of the control bar and anhydrous glucose solution (Figure 2B) and, consequently, glycemic index (GI) [64.20 (CB), 53.20 (PB), 40.40 (MB)] and glycemic load (GL) values [10.9 (CB), 7.9 (PB), 6.1 (MB), *p* < 0.05] followed the same trend (Figure 2C). 

The bars under study are classified based on their GI as moderate = control bar, low = mango bar, and pineapple bar. Therefore, despite no statistical differences being observed, a lower GI for fruit bars compared to CB is reported according to classification.

### 3.3. Enzymatic Inhibition: In Vitro Assay

The pancreatic α-amylase activity inhibition assay was carried out to evaluate the capacity of the phenolic compounds (PCs) present in ethanolic extracts of MB, pineapple PB, CB, acarbose standard, and gallic acid standard, which are shown in Table 2.

The enzymatic inhibition assay was carried out at minute 60, with four different percentages of inhibitor (25, 50, 75, 100). For this case, known concentrations of total soluble polyphenols (TSP) were used, which, for MB, were 46.47 mg/g, PB: 14.09 mg/g, and CB: 5.01 mg/g, which were statistically compared with the controls, acarbose 8 mg, and gallic acid 10 mg.

According to the results (Table 2), it is observed that in the lowest concentration of the inhibitor (25%), the lowest inhibition percentages of pancreatic α-amylase activity are obtained. At this concentration, MB, PB, acarbose, and gallic acid showed higher % of inhibition (16.29 ± 0.23, 15.37 ± 0.05, 15.94 ± 0.01, and 14.27 ± 0.01, respectively) in contrast to CB (4.25 ± 0.1%). In 50 and 75% concentrations of the inhibitors, a tendency was observed regarding the highest inhibition by MB (33.68 ± 0.15 and 49.62 ± 0.1%, followed by PB (31.79 ± 0.31 and 45.93 ± 0.2%), then acarbose and gallic acid, and CB presented the lowest % of inhibition (5.53 ± 0.11 and 8.08 ± 0.34). Also, significant differences were found between the samples used: MB exerted the highest inhibition of α-amylase activity in a 50 and 75% concentration of inhibitor. In the same way, for the concentration of 100% inhibitor, it was observed that MB, PB, acarbose, and gallic acid, unlike CB, managed to exert up to 50% inhibition of α-amylase activity, highlighting MB with better inhibition values.

The intestinal α-glucosidase activity inhibition assay was carried out to evaluate the capacity of the PC present in ethanolic extracts of BM, BP, CB, a standard of acarbose, and gallic acid, as shown in Table 2.

According to the data obtained for the inhibition of α-glucosidase, it is observed that in the case of the 25% and 50% concentration of inhibitor, there are significant differences between the five samples used; PB showed the highest inhibition (27.54 ± 0.8 and 45.31 ± 0.14%, respectively), then MB (25.85 ± 0.37 and 37.46 ± 0.12%), acarbose, gallic acid, and the lowest inhibition by CB (6.84 ± 0.1 and 7.42 ± 0.4%). PB stands out for 75% inhibitor concentration, which inhibits more than 50% of the enzymatic activity (α-glucosidase), while the four remaining samples present less than 50% inhibition. In 100% inhibitor, there are significant differences between the samples used, and it is identified that MB and PB showed statistically the same highest percentage of inhibition (64.97 ± 0.2 and 64.57 ± 0.34%, respectively). The acarbose (62.98 ± 0.61%) and gallic acid (57.02 ± 0.07%) also inhibited more than 50%.

### 3.4. In Silico Assay for α-Amylase and α-Glucosidase

Considering the potential affinity of the identified compounds in MB and PB, the coupling was evaluated considering the kcal/mol values necessary to carry out a potential coupling. The lower the kcal/mol value, the greater the coupling probability. Table 3 shows the values in kcal/mol obtained in the molecular coupling of the different PC (ligands) with α-amylase and α-glucosidase. Acarbose (CID 444254) was considered a positive control, presenting the lowest energy necessary to couple with both enzymes. Kaempferol did not present an energy value with α-amylase; therefore, it is incapable of interacting with the enzyme; in contrast, quercetin has lower total coupling energy than the other phenolic compound (PC). Quercetin´s kcal/mol value is 37.17% higher than the value obtained in acarbose; another compound with a value 37.37% higher than acarbose and 0.2% higher than quercetin was catechin. Butein, naringenin, and ferulic acid have values of 39.50, 39.66, and 39.69%, respectively. Therefore, quercetin and catechin are the main compounds potentially interacting with this enzyme.

In the case of α-glucosidase, the lowest value in kcal/mol is presented with catechin, followed by quercetin, kaempferol, butein, naringenin, and ferulic acid, with values 56.22, 56.55, 57.96, 57.68 and 58.12% higher, respectively. These results indicate that these compounds require more energy to couple with the enzyme α-glucosidase. Thus, the most significant interaction of PC would be with the enzyme α-amylase.

**Figure 3:**
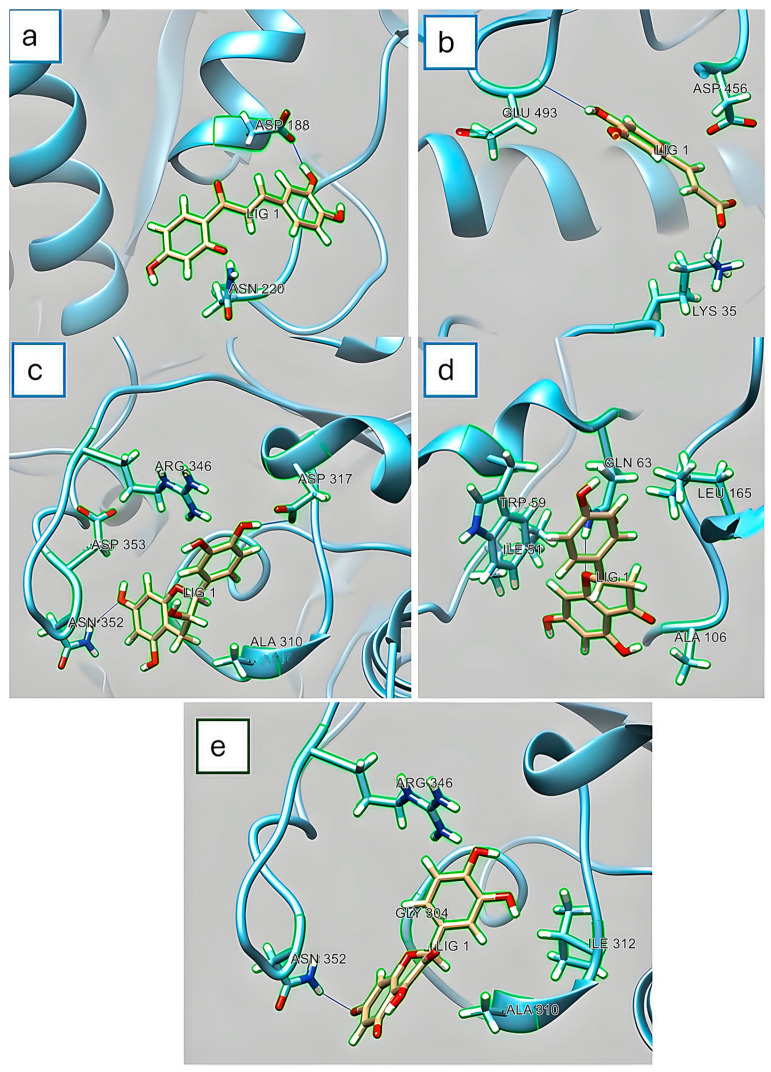
Molecular docking simulation obtained with the lowest energy conformation: α-amylase enzyme. (**a**) α-amylase–butein complex, interactions of aspartic acid (Asp188) and asparagine (Asn220) with LIG 1 (Butein). (**b**) α-amylase–ferulic acid complex, interactions of glutamic acid (Glu493) and lysine (Lys35) with LIG 1 (ferulic acid). (**c**) Complex α–amylase–catechin, interactions of asparagine (Asn352) and aspartic acid (Asp317) with LIG 1 (catechin). (**d**) α–amylase–Naringenin complex, interactions of glutamine (GLN63) with LIG 1 (naringenin). (**e**) α–amylase–quercetin complex, asparagine (Asn352) interactions with LIG 1 (quercetin).

Figure 3 shows the molecular couplings of PC with the α-amylase enzyme. It was identified that the PC present is hydrophilic in different amino acids, which are not responsible for the catalytic activity, for which it is considered that there is an allosteric interaction. This is possible because the active site (substrate binding) of α-amylase is located in a long cleft between the carboxyl terminus of the A and B domains. Specifically, Figure 3a shows a hydrophilic interaction of butein with aspartic acid (Asp188) and asparagine (Asn220) residues of the α-amylase enzyme. Figure 3b presents the α-amylase–ferulic acid complex, with interactions of glutamic acid (Glu493) and lysine (Lys35) with ferulic acid. Figure 3c–e demonstrate the hydrophilic interactions of asparagine (Asn352) and aspartic acid (Asp317) with catechin; the interactions of glutamine (GLN63) with naringenin, and the interactions of the α-amylase–quercetin complex, that is, asparagine, (Asn352) with quercetin, respectively.

**Figure 4:**
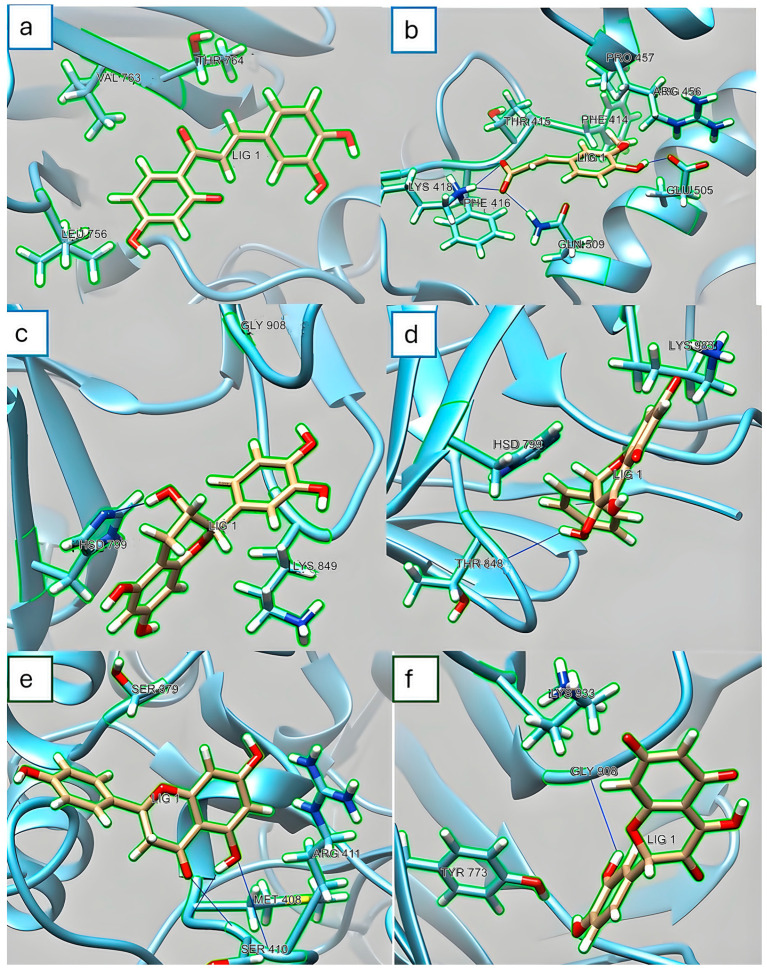
Molecular docking simulation obtained with the lowest energy conformation: α-glucosidase enzyme. (**a**) α-glucosidase–butein complex, interactions valine (val763), leucine (leu756) and threonine (thr764) with lig 1 (butein). (**b**) α-glucosidase–ferulic acid complex, interactions of phenylalanine (phe416), glutamic acid (glu505), glutamine (gln509) and lysine (lys418) with lig 1 (ferulic acid). (**c**) α-glucosidase–catechin complex, interactions of histidine (hsd799) with lig 1 (catechin). (**d**) α-glucosidase–kaempferol complex, threonine (thr848) interactions with lig 1 (kaempferol). (**e**) α-glucosidase–naringenin complex, interactions of serine (ser410) and methionine (met408) with lig 1 (naringenin). (**f**) α-glucosidase–quercetin complex, interactions of glycine (gly908) with lig 1 (quercetin).

Regarding the results with the α-glucosidase enzyme, Figure 4 shows the molecular docking simulation obtained with the different ligands (PC). Figure 4a shows hydrophilic interactions of butein with valine (Val763), leucine (Leu756), and threonine (Thr764) residues in the α-glucosidase enzyme. In Figure 4b, it is observed that ferulic acid interacts with residues of phenylalanine (Phe416), glutamic acid (Glu505), glutamine (Gln509), and lysine (Lys418). In the case of catechin (Figure 4c), an interaction was noticed in residues of histidine (Hsd799), Gly908, and Lys849. In Figure 4d, it is observed that kaempferol has interaction in residues of Thr848, His798, and Lys938. Figure 4e shows that naringenin interacts with serine and methionine residues (Ser410 and Met408). Figure 4f indicates that quercetin shows interactions in residues of glycine (Gly908), Tyr773, and Lys833. However, considering the kcal/mol values detected for these ligands, more incredible energy is required for these interactions. 

## 4. Discussion

As expected, the chemical composition of the evaluated bars showed heterogeneity and significant differences between them due to the raw material used for their production (pineapple or mango). Mangoes are rich in minerals such as calcium, magnesium, phosphorus, sodium, and iron and poor ~0.51 g/100 g in protein, while pineapple is rich in sodium, potassium, and iron and poor ~0.54 g/100 g in protein [33,34]. Therefore, the ashes, protein, and moisture content varied between fruit bars.

Although carbohydrates (CHOs) were the major component of the three bars, MB and PB showed a lower content versus CB, as well as lower content of lipids and remarkably higher dietary fiber and TSP content, which may make them a healthier snack option than those usually available on the current market. It has been shown that high consumption of dietary fiber from dehydrated fruits can positively affect blood pressure and glycemia and ease glycemic regulation in T2DM. It may reduce the risk of cardiovascular disease, mainly due to their richness in associated phenolic compounds (PCs) [35].

Previously, Hernández-Maldonado et al. [17] reported in MB the presence of gallic acid, coumaric acid, ferulic acid, caffeic acid, quercetin, mangiferin, and, in the case of PB, reported content of hydroxybenzoic acids, hydroxycinnamic acids, flavonoids, and hydrolyzable tannins [16]. In this way, an interesting report indicates that the consumption of dehydrated mango snack bars rich in PC and dietary fiber favors the growth of beneficial colonic bacterial groups (*Faecalibacterium*, *Roseburia*, *Eubacterium*, *Fusicatenibacter*, *Holdemanella*, *Catenibacterium*, *Phascolarctobacterium*, *Buttiauxella*, *Bifidobacterium*, *Collinsella*, *Prevotella* and *Bacteroides*), leading to reduced metabolic dysbiosis [36]. 

Regarding the in vivo assay of the glycemic response, the biological response observed in participants (A) could be partially explained by the fact that MB and PB had almost the same carbohydrate (sugars + starch: ~50 g.100 g^−1^) and total dietary fiber content (~30 g.100 g^−1^), statistically different from the control bar (~50 g and ~7.100 g^−1^, respectively). The soluble dietary fiber in a portion of food provides texture and viscosity; these influences inhibit the absorption of macronutrients and some blood lipids and can regulate postprandial glucose levels since gastric emptying is delayed [27]. From a mechanistic standpoint, the maximum glycemic peak often occurs within the first 30 min, most likely related to the intestinal absorption of free and rapidly hydrolyzed (from digestible polysaccharides) glucose more than a systemic hormonal control. 

High-GI foods often exhibit a sharp glycemic increment between 0 and 30 followed by a rapid fall between 12 and 30 min and a high iAUC that sometimes includes AUC below basal levels in the last minutes of the 2 h period of testing [27]. The lack of statistical differences between 0 and 60 min (high interindividual variability) and the subtle differences between 60 and 120 min between samples (Figure 2A) preliminary suggests a luminally controlled glucose release from digestible polysaccharides. However, a differential insulinemic response cannot be ruled out. Such phenomena could result from controlled enzyme inhibition, modest differences in soluble/insoluble dietary fiber, or the inhibition of glucose transport [23]. 

It is important to mention that in normoglycemic subjects, the postprandial glycemic response to fifty grams of available carbohydrates, either coming from standard oral glucose or a composed food, tends to return to pre-prandial values after 2 h, exhibiting a trapezoid-like behavior [26]. The iAUC calculated with the trapezoid rule is quite precise in discriminating high (GI ≥ 70), moderate (GI 56–69), and low (GI ≤ 55) glycemic foods [37]; however, foods are rarely eaten alone or in 50 g available carbohydrate servings, so GI values are adjusted for serving size and referred to as glycemic load (GL) values [24]. Although a direct causal relationship cannot be established for specific foods such as all those evaluated here, consuming low-GI and low-GL diets abundant in low-GI/GL foods, such as mango and pineapple bars, has been associated with a reduction in the risk of many cardiometabolic diseases [38].

Furthermore, several studies have linked the presence of phenolic compounds in the food matrix with a lower GI, attributed to different mechanisms: acting as inhibitors of digestive enzymes, affecting the absorption of glucose in the intestine, regulating the function of the β cells of the pancreas, promoting the transport of glucose in the bloodstream, and regulating the production of glucose through the gluconeogenesis pathway [39].

Soluble dietary fiber (SDF) comprises pectins and other non-starch polysaccharides in mango, pineapple pulp, and peel. The inclusion of peels in the bars adds cellulose, lignin, and hemicellulose (arabinoxylans and arabinogalactans), which are important constituents of insoluble dietary fiber (IDF) in fruits [40]. The relevance of dietary fiber goes beyond influencing glycemic index (GI) and various health outcomes. Research indicates that dietary fiber improves satiety and intestinal mobility and reduces glucose absorption because of the viscosity that impairs the SDF. Their supplementation during pregnancy can prevent gestational diabetes mellitus (GDM) and preterm birth in women with a high triglyceride–glucose (TyG) index [41]. Additionally, high-fiber snacks with a medium GI may improve glucose and insulin homeostasis [42]. Studies also suggest that GI and dietary fiber intake are associated with the risk of renal cell carcinoma, with fiber intake showing an inverse association while GI shows a positive association [43].

Based on the data obtained for inhibition of both α-amylase and α-glucosidase, it is verified that PCs such as acarbose can become very effective inhibitors. For this reason, they can be considered a sustainable strategy for preventing hyperglycemia [44]. It has been identified that resveratrol acts non-competitively at a concentration of 3.62 µg/mL against α-glucosidase, with which a constant intake of at least 0.5 g/day can exert a therapeutic effect in T2DM patients [45,46].

In that respect, the results obtained when evaluating the effect of fruit bar extracts can be attributed to the presence of compounds such as quercetin, ferulic acid, and gallic acid, which are potential inhibitors of α-amylase and α-glucosidase and can be released in the gastric fraction [17]. It is important to point out that the effects exerted by the extracts of MB and PB are comparable to those of acarbose without the adverse effects attributed to this medication, such as gastrointestinal disturbances, flatulence, abdominal pain, and even diarrhea [47].

About the findings in the molecular docking assay, it is worth mentioning that the active site of α-amylase enzyme contains three catalytic residues, Asp179, Glu204, and Asp289, and a long gap is found between the carboxyl terminus of A and domain B [48,49]. In this sense, Acarbose has been shown to bind at two sites in α-amylase (Trp276 and Trp277), in addition to interacting with Asp87, His92, Arg177, Asp179, Glu204, His288, and Asp289 [50].

Taha et al. [51] reported interactions (conventional hydrogen bonds) of acarbose in the amino acids His201, Arg195, Glu233, Asp300, His299, Trp59, Gln63, Thr163, His305, and Gly306 in α-amylase (1B2Y), for which its high reactivity is due to a large amount of OH groups in this compound. Also, the importance of the hydroxyl groups of polyphenols in the interaction with amino acid residues in the active site of α-amylase has been reported. Molecular coupling analysis previously suggested that eliminating hydroxyl groups from polyphenols can decrease the inhibition effect [52,53]. Quercetin has five hydrogen donor bonds and seven acceptors, while catechin has the same number of hydrogen donors but only six acceptors. Both molecules have one rotatable bond, and what changes is the topological area of the polar surface; while catechin has 110 Å^2^, quercetin has 127 Å^2^. This could explain the greater affinity that these compounds have for this enzyme, and compared to Acarbose, which has 14 hydrogen donors and 19 acceptors, its polar surface topological area is 321 Å^2^; this value is approximately three times higher than those of quercetin and catechin. This could explain the kcal/mol values presented by both compounds. 

Rasouli et al. [54], studied 27 compounds and found that only 5 (catechin, hesperetin, kaempferol, silibinin, and pelargonidin) are potential molecules to interact with the enzyme’s active site. Considering the results obtained from the enzymatic activity in vitro, this could explain the controlled inhibition of the hypoglycemic activity.

According to previous scientific studies on molecular coupling and binding interactions with PC and digestive enzymes (α-amylase, α-glucosidase), most revealed hydrogen bonding with active site residues. Catechin, naringenin, and ferulic acid bound to α-glucosidase at residues Val763, Leu756, Arg319, Thr376, Arg387, Trp388, Gln390, and kaempferol at Tyr62, Gln63, Val107, Leu162, His299, and Glu233 at the catalytic site of α-glucosidase [55]. In addition, Hua et al. [56] have shown that kaempferol participated in the hydrogen bonding of α-glucosidase with His201, Glu233, Asp197, Gln63, and Trp59 with kaempferol monoglycoside. Naringenin forms hydrogen bonds and hydrophobic interaction with α-glucosidase in Asp307, Gly309, Ser311, Pro312, Val319, Pro320, Asp325, and Ala329 for complex stability [57]. It has been demonstrated that α-glucosidase’s inhibitory capacity is comparable to Acarbose because of its binding interaction with the active site [58]. These results show that the main activity of the PC is initially the allosteric inhibition of α-amylase, which is reflected in a decrease in the glycemic response, which was observed in the results in enzymatic activity.

## 5. Conclusions

The synergy between the evaluated bars’ dietary fiber content and phenolic compounds showed a positive effect that favors a lower postprandial glycemic response than the control bar. The employment of ethanolic extracts of MB and PB has an inhibitory effect on the activity of α-amylase and α-glucosidase; values greater than 60% inhibition were presented using a concentration of 100% extract of PC, which showed a competitive inhibition mechanism for α-amylase and a non-competitive mechanism for α-glucosidase compared to the controls used. Naringenin and ferulic acid exhibited more favorable interaction energies against α-amylase and α-glucosidase, which shows their high inhibitory power against these enzymes governed by an allosteric regulation mechanism.

It is crucial to thoroughly utilize and assess a food item that serves as a nutritious snack. This investigation enabled us to present a more extensive view of the potential beneficial impacts on health, particularly in glycemic regulation, of incorporating minimally processed food choices into our daily diet. Consequently, we aim to enhance public health and diminish food wastage, thus moving towards a more sustainable dietary pattern. This study paves the way for further research to develop functional foods fortified with dietary fiber and phenolic compounds, leveraging these natural elements to manage blood glucose levels.

## Figures and Tables

**Table 1:** Chemical composition of mango bar, pineapple bar, and control bars by portion size (g/30 g).

Parameter	Mango Bar	Pineapple Bar	Control Bar
Moisture	10.23 ± 0.03 a	11.54 ± 0.01 b	13.15 ± 0.01 c
Ashes	2.08 ± 0.03 a	1.93 ± 0.00 b	1.24 ± 0.00 c
Protein	2.41 ± 0.06 a	2.73 ± 0.04 b	1.63 ± 0.04 c
Lipids	1.09 ± 0.00 a	0.19 ± 0.00 b	4.64 ± 0.04 c
Carbohydrates	15.06 ± 0.2 a	14.88 ± 0.2 a	17.01 ± 0.1 b
Total dietary fiber	9.55 ± 0.2 a	7.37 ± 0.3 b	1.96 ± 0.1 c
Total phenolics (mg GAE/g)	46.47 ± 0.1 a	14.09 ± 0.0 b	5.01 ± 0.2 c

Values are the mean ± standard deviation (*n* = 3). Lowercase letters represent significant. Differences per row (*p* < 0.05) using a Fisher LSD test for means comparison.

**Table 2:** Enzymatic inhibition of α-amylase α-glucosidase by ethanolic extracts of the control bar, mango bar, pineapple bar, acarbose, and gallic acid at t = 60 min.

Concentration	Control Bar	Mango Bar	Pineapple Bar	Acarbose	Gallic Acid
α-amylase					
25%	4.25 ± 0.1 a	16.29 ± 0.23 b	15.37 ± 0.05 b	15.94 ± 0.01 b	14.27 ± 0.01 b
50%	5.53 ± 0.11 a	33.68 ± 0.15 b	31.79 ± 0.31 c	29.77 ± 0.24 c	27.27 ± 0.07 d
75%	8.08 ± 0.34 a	49.62 ± 0.1 b	45.93 ± 0.2 c	42.24 ± 0.01 d	38.38 ± 0.17 e
100%	9.83 ± 0.5 a	61.44 ± 0.35 b	59.37 ± 0.1 b	54.23 ± 0.6 c	52.39 ± 0.44 c
α-glucosidase					
25%	6.84 ± 0.1 a	25.85 ± 0.37 b	27.54 ± 0.8 c	24.21 ± 0.04 d	20.57 ± 0.2 e
50%	7.42 ± 0.4 a	37.46 ± 0.12 b	45.31 ± 0.14 c	39.84 ± 0.27 d	34.24 ± 0.31 e
75%	10.15 ± 0.2 a	48.66 ± 0.51 b	50.82 ± 0.6 c	47.04 ± 0.87 b	45.51 ± 0.16 d
100%	12.46 ± 0.08 a	64.97 ± 0.26 b	64.57 ± 0.34 b	62.98 ± 0.61 c	57.02 ± 0.07 d

Values are the mean ± standard deviation (*n* = 3). Lowercase letters represent significant differences per row *(p* < 0.05) using a Fisher LSD test for means comparison.

**Table 3:** Affinity of the molecular coupling of the best α-amylase-ligand and α-glucosidase-ligand enzyme complexes.

Bind	α-Amylase	α-Glucosidase
	(Kcal/mol)	
Ferulic acid	−1692.8904	−2760.3513
Buteine	−1690.6622	−2752.7178
Catechin	−1664.7638	−2727.2085
Kaempferol	-	−2733.0417
Naringenin	−1692.5985	−2757.674
Quercetin	−1662.3651	−2729.2283
Acarbose	−1211.8814	−1745.7192

Data expressed as the total energy of the binding complex (Fullfitnes). No present energy value.

## Data Availability

The original contributions presented in the study are included in the article and Supplementary Materials, further inquiries can be directed to the corresponding author.

## Supplementary Materials

The following supporting information can be downloaded at: https://www.mdpi.com/article/10.3390/foods13142258/s1, Figure S1. Fruit, mango and pineapple bars; Figure S2. Capillary puncture and glucose determination.
