# Supplementary material for: In Vivo Glycemic Response of Fruit-Based Mango (Mangifera indica) and Pineapple (Ananas comosus) Bars in In Vitro and In Silico Enzyme Inhibitory Effects Studies
Source: Foods. 2024 Jul 17;13(14):2258. doi: 10.3390/foods13142258 (PMC11276214; doi:10.3390/foods13142258)
Supplement: Supplementary file 1 [file foods-13-02258-s001.zip › foods-3094253-supplementary.pdf]

Universidad Autónoma de Ciudad Juárez
Instituto de Ciencias Biomédicas
Cuerpo Académico en Salud Comunitaria

CONSENTIMIENTO INFORMADO

Nombre del participante: ____

Se te está invitando a para participar en el estudio denominado **“Evaluación del índice glicémico *in vivo* de barras de mango (*Mangifera indica L.*) y piña (*Ananas comosus L.*)”**; el consentimiento escrito e informado es un requerimiento ético de cualquier estudio clínico experimental. Antes de decidir si participas o no, debes conocer y comprender cada uno de los apartados que adelante se describe de forma escrita y que te son explicados por el personal del estudio; siéntete con absoluta libertad de preguntar sobre cualquier aspecto que te ayude a aclarar todas tus dudas.

I. Justificación y objetivo(s) del estudio

Los mexicanos como tú y yo, poseemos un alto riesgo de padecer enfermedades crónicas no transmisibles (ECNT). Muchas de estas enfermedades se deben al estilo de vida dentro de las cuales se incluyen, el sedentarismo y el consumo de alimentos densamente energéticos como lo son las barras de cereal con gran cantidad de azucares simples y pobres en fibra (**Figure S1a**). Para mejorar la salud y prevenir ECNT se recomienda el consumo de alimentos con menos azúcar adicionada y con un mejor perfil nutrimental como es el caso de las “**Barras de frutas tropicales**” (**Figure S1b**). Particularmente, el mango y la piña son dos frutas tropicales con un perfil nutrimental/funcional ideal para la formulación de alimentos convenientes y portables. Además, estudios realizados dentro de nuestra universidad los respaldan como fuentes únicas de antioxidantes necesarios para la prevención y control de diversas ECNT incluidas a la diabetes mellitus.

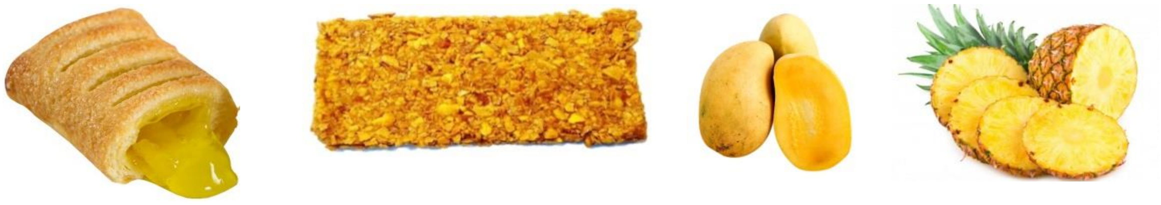

a **B** **c** **d**

Figure S1. Barras de fruta, mango y pina

La intención del presente estudio es la de proveer de información científicamente validada que permita reposicionar el consumo de barras de frutas preparadas con pulpa de mango o piña sin azúcar añadida, como una herramienta para prevenir las ECNT de la población mexicana y muy en particular en jóvenes como tú.

II. **Características del estudio**

A lo largo del estudio (3 sesiones/ ~2.0h/ cada 3er día, participaras en un protocolo experimental para evaluar la respuesta glicémica post-ayuno (postprandial) de tres barras (barra a y barras preparadas con c y d, **Figure S1**) El estudio se realizará en el Laboratorio de Fisiología del Ejercicio de la Universidad Autónoma de Ciudad Juárez y estará dirigido por el Dr. Abraham Wall Medrano, responsable del proyecto y con la colaboración de estudiantes de pre y posgrado.

Durante este tiempo se te pedirá que atiendas a cada sesión en ayuno de al menos 8 horas previas y en la primera sesión se te realizara una evaluación antropométrica. En todas las sesiones se te tomarán muestras de sangre por punción capilar (**Figure S2**) antes (tiempo cero) y posterior (30, 60, 90 y 120 minutos) al consumo de alimento asignado de forma aleatoria (doble ciego).

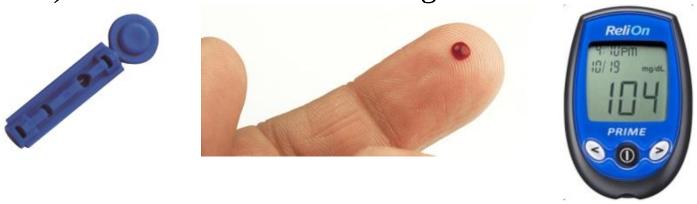

a **b** **c**

Figure S2. Punción capilar y determinación de glucosa

Durante todos los procedimientos serás supervisado continuamente por el personal que te atiende mediante la medición de tus signos vitales, todo lo anterior en apego a las normas de salud vigentes.

III. Riesgos asociados al estudio

Por la naturaleza del proyecto NO EXISTE ningún riesgo de salud inminente durante la etapa post-ayuno pus solo se toman muestras sanguíneas. Sin embargo, es posible que tras la ingestión de los alimentos se experimenten molestias producto de su consumo en un lapso muy corto (10 min). En tal caso, el personal con entrenamiento en primeros auxilios te ayudara.

IV. Aclaraciones

- Tu decisión de participar en el estudio es completamente voluntaria.
- Durante el estudio puedes solicitar información actualizada sobre el mismo al investigador responsable.
- No habrá ninguna consecuencia desfavorable en caso de no aceptar la invitación.
- Para evitar o disminuir posibles riesgos a su salud, el personal del laboratorio supervisará de manera constante diversos parámetros fisiológicos y signos vitales.
- En caso de que desarrolles algún efecto adverso secundario no previsto tienes derecho a no continuar en el estudio y en caso extremo a una indemnización, siempre y cuando los efectos adversos sean a consecuencia de la realización del protocolo experimental que te fue explicado.

- Si consideras que no hay dudas ni pregunta acerca de tu participación, puedes si así lo deseas firmar esta carta de consentimiento.
- La información recabada solo será útil para los fines de la investigación y se garantiza el anonimato de todos los participantes. La confidencialidad de la información recabada seguirá los lineamientos estipulados por el Reglamento de la Ley General de Salud en materia de Investigación para la salud (Artículos 13 y 16), Ley de Información Estadística y Geográfica (Artículos 38 y 42) y el comité de ética de la UACJ.
- Recibirás una copia firmada y fechada de este formulario de consentimiento.

V. Consentimiento

He leído y comprendido la información anterior y mis preguntas han sido respondidas de manera satisfactoria. He sido informado y convengo en participar en el estudio. Una vez concluida la sesión de preguntas y respuestas, se procedió a firmar el presente documento.

______________________________	______________________________
Firma del Investigador	Fecha:
______________________________	______________________________
Firma del Participante	Fecha:

Responsable del estudio: **Dr. Abraham Wall Medrano (CA en Salud Comunitaria)**

Yo, **______________________________________**, integrante del equipo de investigación, he explicado la naturaleza y los propósitos del estudio. Le he explicado a cerca de los beneficios y posibles perjuicios que implica su participación. He contestado a las preguntas en la medida de lo posible y he preguntado si tiene alguna duda. Acepto que he leído y conozco la normatividad correspondiente para realizar investigaciones con seres humanos y me apego a ellas.
